# Characterization and Sequencing of a Genotype VIId Newcastle Disease Virus Isolated from Laying Ducks in Jiangsu, China

**DOI:** 10.1128/genomeA.01412-15

**Published:** 2015-12-03

**Authors:** Mei Liu, Xinyue Shen, Xu Cheng, Jianmei Li, Yabin Dai

**Affiliations:** a Poultry Institute, Chinese Academy of Agricultural Sciences, Yangzhou, Jiangsu, China; b Jiangsu Co-Innovation Center for Prevention and Control of Important Animal Infectious Diseases and Zoonoses, Yangzhou, Jiangsu, China

## Abstract

We report here the complete genome sequence and biological characterization of a virulent Newcastle disease virus (NDV) strain, NDV/duck/Jiangsu/JSD0812/2008, isolated from laying ducks in Jiangsu Province, China. The genome is 15,192 nucleotides in length and is classified in subgenotype VIId of genotype VII, class II.

## GENOME ANNOUNCEMENT

Newcastle disease (ND), caused by virulent Newcastle disease virus (NDV), is an acute, highly contagious, and fatal viral disease of birds (1). NDV is a member of the genus *Avulavirus* within the family *Paramyxoviridae*, subfamily *Paramyxovirinae* (2). Based on sequence analysis of the fusion (F) gene, NDV strains are divided into two distinct classes (I and II). Almost all virulent NDV strains isolated from wild and domestic birds belong to class II, which can be further subdivided into at least 18 genotypes from I to XVIII (3).

NDV is able to infect at least 241 species from 27 of the 50 orders of birds. Waterfowl, such as ducks and geese, are generally considered to be natural reservoirs or carriers of NDV and may show few or no clinical signs when infected with viruses, even those most virulent for chickens (1). However, ND outbreaks in domestic waterfowl have frequently been reported in the past decade (4).

There were several outbreaks of ND in egg-laying duck farms in Jiangsu, China, in late 2008. In the affected flocks, egg production sharply declined by about 70%, morbidity was about 80%, and mortality varied from 30% to 50%. The diseased birds showed diarrhea and nervous signs, and the dead birds mainly manifested by focal hemorrhage, necrosis of the intestinal mucosa, and congestion and hemorrhage of the ovarian follicles. NDV/duck/Jiangsu/JSD0812/2008 (JSD0812) was isolated from a liver-spleen suspension in 10-day-old embryonated specific-pathogen-free (SPF) chicken eggs.

Strain JSD0812 was classified as velogenic NDV, with a mean death time (MDT) of 54.6 h, an intracerebral pathogenicity index (ICPI) of 1.75, and an intravenous pathogenicity index (IVPI) of 2.68 (5). These characteristics were consistent with the presence of a polybasic amino acid motif (^112^RRQKRF^117^) at the fusion protein cleavage site, which is considered a major molecular determinant of virulence for NDV (6).

Animal experiments demonstrated that JSD0812 was highly pathogenic for chickens, geese, and ducks (4, 5). Further studies confirmed that the pathogenicity of JSD0812 in ducks was closely related to age, breed, dose, and inoculation route. Clinical manifestations of the infected ducks were primarily neurologic, including twisting of head and neck, lack of muscular coordination, circling, and muscular tremors. Some surviving birds showed poor subsequent growth or maldevelopment of one or both legs (7).

The complete genome of JSD0812 was amplified by 14 pairs of overlapping oligonucleotide primers and found to be 15,192 nucleotides (nt) long. Phylogenetic analysis of the complete genome classified this strain into class II, genotype VII, subgenotype VIId. The highest homology (99.1%) was found with NDV/Muscovy duck/China(Fujian)/FP1/02 (GenBank accession no. FJ872531), which was also highly pathogenic for ducks (8), while only 82.1% was found with the LaSota strain (GenBank accession no. AF077761).

The data presented here will aid future research in the epidemiology and molecular pathogenesis of NDV and suggest that prevention of ND in ducks should receive due attention and further research.

### Nucleotide sequence accession number.

The complete genome sequence of NDV/duck/Jiangsu/JSD0812/2008 strain has been deposited in GenBank under the accession no. GQ849007. The version described in this paper is the first version.
